# Parameters of proteome evolution from histograms of amino-acid sequence identities of paralogous proteins

**DOI:** 10.1186/1745-6150-2-32

**Published:** 2007-11-26

**Authors:** Jacob Bock Axelsen, Koon-Kiu Yan, Sergei Maslov

**Affiliations:** 1 Center for Models of Life, Niels Bohr Institute, Blegdamsvej 17, DK-2100, Copenhagen Ø, Denmark; 2 Department of Condensed Matter Physics and Materials Science, Brookhaven National Laboratory, Upton, New York 11973, USA; 3 Department of Physics and Astronomy, Stony Brook University, Stony Brook, New York 11794, USA

## Abstract

**Background:**

The evolution of the full repertoire of proteins encoded in a given genome is mostly driven by gene duplications, deletions, and sequence modifications of existing proteins. Indirect information about relative rates and other intrinsic parameters of these three basic processes is contained in the proteome-wide distribution of sequence identities of pairs of paralogous proteins.

**Results:**

We introduce a simple mathematical framework based on a stochastic birth-and-death model that allows one to extract some of this information and apply it to the set of all pairs of paralogous proteins in *H. pylori*, *E. coli*, *S. cerevisiae*, *C. elegans*, *D. melanogaster*, and *H. sapiens*. It was found that the histogram of sequence identities *p *generated by an all-to-all alignment of all protein sequences encoded in a genome is well fitted with a power-law form ~ *p*^-*γ *^with the value of the exponent *γ *around 4 for the majority of organisms used in this study. This implies that the intra-protein variability of substitution rates is best described by the Gamma-distribution with the exponent *α *≈ 0.33. Different features of the shape of such histograms allow us to quantify the ratio between the genome-wide average deletion/duplication rates and the amino-acid substitution rate.

**Conclusion:**

We separately measure the short-term ("raw") duplication and deletion rates $r_{\text{dup}}^{*}$, $r_{\text{del}}^{*}$ which include gene copies that will be removed soon after the duplication event and their dramatically reduced long-term counterparts *r*_dup_, *r*_del_. High deletion rate among recently duplicated proteins is consistent with a scenario in which they didn't have enough time to significantly change their functional roles and thus are to a large degree disposable. Systematic trends of each of the four duplication/deletion rates with the total number of genes in the genome were analyzed. All but the deletion rate of recent duplicates $r_{\text{del}}^{*}$ were shown to systematically increase with *N*_genes_. Abnormally flat shapes of sequence identity histograms observed for yeast and human are consistent with lineages leading to these organisms undergoing one or more whole-genome duplications. This interpretation is corroborated by our analysis of the genome of *Paramecium tetraurelia *where the *p*^-4 ^profile of the histogram is gradually restored by the successive removal of paralogs generated in its four known whole-genome duplication events.

## Open peer review

This article was reviewed by Eugene Koonin, Yuri Wolf (nominated by Eugene Koonin), David Krakauer, and Eugene Shakhnovich.

## Background

The recent availability of complete genomic sequences of a diverse group of living organisms allows one to quantify basic mechanisms of molecular evolution on an unprecedented scale. The part of the genome consisting of all protein-coding genes (the full repertoire of its proteome) is at the heart of all processes taking place in a given organism. Therefore, it is very important to understand and quantify the rates and other parameters of basic evolutionary processes shaping thus defined proteome. The most important of those processes are:

• Gene duplications that give rise to new protein-coding regions in the genome. The two initially identical proteins encoded by a pair of duplicated genes subsequently diverge from each other in both their sequences and functions.

• Gene deletions in which genes that are no longer required for the functioning of the organism are either explicitly deleted from the genome or stop being transcribed and become pseudogenes whose homology to the existing functional genes is rapidly obliterated by mutations.

• Changes in amino-acid sequences of proteins encoded by already existing genes. This includes a broad spectrum of processes including point substitutions, insertions and deletions (indels), and transfers of whole domains either from other genes in the same genome or even from genomes of other species.

The BLAST (blastp) algorithm [1] allows one to quickly obtain the list of pairs of paralogous proteins encoded in a given genome whose amino-acid sequences haven't diverged beyond recognition. The set of their percentage identities (PIDs) is a dynamic entity that changes due to gene duplications, deletions, and local changes of sequences. Duplication events constantly create new pairs of paralogous proteins with PID = 100%, while subsequent substitutions, insertions and deletions result in their PID drifting down towards lower values. A paralogous pair disappears from this dataset if one of its constituent genes is deleted from the genome, becomes a pseudogene, or when the PID of the pair becomes too low for it to pass the E-value cutoff of the algorithm. Thus the PID histogram contains a valuable if indirect information about past duplications, deletions, and sequence divergence events that took place in the genome. In what follows we propose a mathematical framework allowing one to extract some of this information and quantify the average rates and other parameters of the basic evolutionary processes shaping protein-coding contents of a genome.

The list of all paralogous pairs generated by the all-to-all alignment of protein sequences encoded in a given genome is generally much larger than the list of pairs of sibling proteins created by individual duplication events. For example, a family consisting of *F *paralogous proteins contributes up to *F*(*F *- 1)/2 pairs to the all-to-all BLAST output, while not more than *F *- 1 of these pairs connect the actual siblings to each other. The identification of the most likely candidates for these "true" duplicates is in general a rather complicated task which involves reconstructing the actual phylogenetic tree for every family in a genome. This goes beyond the scope of this study, where we employ a much simpler (yet less precise) Minimum Spanning Tree algorithm to extract a putative non-redundant subset of true duplicated (sibling) pairs.

The idea of quantifying evolutionary parameters using the histogram of some measure of sequence similarity of duplicated genes in itself is not new. It was already discussed by Gillespie (see [2] and references therein) and later applied [3] to measure the deletion rate of recent duplicates. There are two important differences between our methods and those of the Ref. [3]:

• We use relatively slow changes in amino-acid sequences of proteins as opposed to much faster silent substitutions of nucleotides used in the Ref. [3]. This allows to dramatically extend the range of evolutionary times amenable to this type of analysis.

• In addition to PID distributions in the non-redundant set of true duplicated pairs used in the Ref. [3] we also study that in the highly redundant set of all paralogous pairs detected by BLAST. It turned out that both these distributions contain important and often complimentary information about the quantitative dynamics of the underlying evolutionary process. The shape of the latter (all-to-all) histogram is to a first approximation independent of duplication and deletion rates and thus it allows us to concentrate on fine properties of amino-acid substitution.

The central results of our analysis are:

• The middle part of the PID histogram of all paralogous pairs detected by BLAST is well described by a powerlaw functional form with a nearly universal value of the exponent *γ *≃ -4 observed in a broad variety of genomes. Our mathematical model relates this exponent to parameters of intra-protein variability of sequence divergence rates.

• The upper part of the PID histogram corresponding to recently duplicated pairs (PID>90%) deviates from this powerlaw form. It is exactly this subset of paralogous pairs that was extensively analyzed in Ref. [3]. This feature is consistent with the picture of frequent deletion of recent duplicates proposed in Ref. [3].

• The analysis of various features of the PID histogram of all paralogous pairs and that of a subset consisting of true duplicated (sibling) pairs allows us to quantify both the long-term average duplication and deletion rates in a given genome as well as a dramatic increase in those rates for recently duplicated genes.

• Abnormally flat PID histograms observed for yeast and human are consistent with lineages leading to these organisms undergoing one or more Whole-Genome Duplications (WGD). This interpretation is corroborated by the genome of *Paramecium tetraurelia *where the PID^-4 ^profile of the sequence identity histogram is gradually restored by the successive removal of paralogs generated in its four known WGD events.

• Applying the same methods to large individual families of paralogous proteins allows one to study the variability of evolutionary parameters within a given genome. It is shown that larger or slower evolving families are characterized by higher inter-protein variability of amino-acid substitution rates.

## Results

### Distribution of sequence identities of all paralogous pairs in a genome

**Figure 1:**
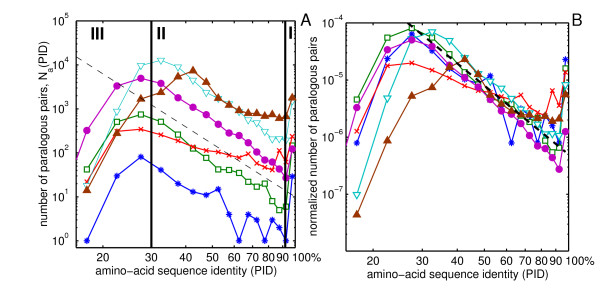
**Histogram of all amino-acid sequence identities**. The histogram *N*_*a*_(*p*) (panel A) and the normalized histogram *n*_*a*_(*p*) = *N*_*a*_(*p*)/[*N*_genes_(*N*_genes _- 1)/2] (panel B) of amino acid sequence identities *p *for all pairs of paralogous proteins in complete genomes of *H. pylori *(blue stars), *E. coli *(green open squares), *S. cerevisiae *(red crosses), *C. elegans *(cyan open triangles), *D. melanogaster *(magenta filled circles) and *H. sapiens *(brown filled triangles). The dashed line is a power-law *p*^-4^. Note the logarithmic scale of both axes. Vertical lines separate regions I, II and III described in the text.

We studied the distribution of Percent Identity (PID) of amino acid sequences of all pairs of paralogous proteins in complete genomes of bacteria *H. pylori*, *E. coli*, a single-celled eukaryote *S. cerevisiae*, and multi-cellular eukaryotes *C. elegans*, *D. melanogaster*, and *H. sapiens*. Every protein sequence contained in a given genome was attempted to be aligned with all other sequences in the same genome using the blastp algorithm [1]. To avoid including pairs of multidomain proteins homologous over only one of their domains we have filtered the data by only keeping the pairs in which the length of the aligned region constitutes at least 80% of the length of the longer protein. Infrequent spurious alignments between different splicing variants of the same gene or between proteins listed in the database under several different names were dropped from our final dataset. The exact details of our procedure are described in the Methods section. Fig. 1A shows the histogram *N*_*a*_(*p*) of amino-acid sequence identities (PIDs) *p *of *all pairs *of paralogous proteins encoded in different genomes. The *p*-dependence of these histograms has three distinct regions I, II, III.

• *Region I*: There is a sharp and significant upturn in the PID histogram above roughly 90–95% compared to what one expects from extrapolating *N*_*a*_(*p*) from lower values of *p*. Apparently the constants (or possibly even mechanisms) of the dynamical process shaping *N*_*a*_(*p*) are different in this region.

• *Region II*: This region covers the widest interval of PIDs 30% <*p *< 90%. *N*_*a*_(*p*) in this region can be approximated by a power-law form of *p*^-*γ *^with *γ *≈ 4 (shown as a dashed line in Fig. 1A.) The best fits to the power-law form in the Region II are listed in Table 1 and (with the exception of yeast and human) they fall in the 3 – 5 range. The near-universality of the shape of the PID histogram is perhaps best illustrated by an approximate collapse of PID histograms in different genomes when they are normalized by the total number *N*_genes_(*N*_genes _- 1)/2, of all (both paralogous and non-paralogous) gene pairs in the genome (Fig. 1B).

**Table 1:** Deletion and duplication rates. The first column contains the name of the organism, the second column – *N*_genes_, the number of genes in its genome, the third column is the value of the exponent *γ *in the best fit with *p*^-*γ *^to *N*_*a*_(*p*) in the region II. The fourth, fifth, sixth and seventh columns are correspondingly the ratios $r_{\text{dup}}^{*}/\bar{\mu }$, *r*_dup_/$\bar{\mu }$, *r*_del_/$\bar{\mu }$, and $r_{\text{del}}^{*}/\bar{\mu }$ defined and measured as described in the text.

**Organism**	Proteome size	*γ*	$r_{\text{dup}}^{*}/\bar{\mu }$	*r*_dup_/$\bar{\mu }$	*r*_del_/$\bar{\mu }$	$r_{\text{del}}^{*}/\bar{\mu }$
*H. pylori*	1590	3.1	0.73	0.032	0.16	67
*E. coli*	4288	4.4	1.37	0.038	0.10	64
*S. cerevisiae*	5885	1.8	1.61	0.24	0.24	27
*C. elegans*	19099	4.2	3.16	0.27	0.37	41
*D. melanogaster*	14015	4.4	0.35	0.084	0.22	30
*H. sapiens*	25319	2.4	2.82	0.85	0.16	19

• *Region III*: In this region *p *< 25 – 30% the histogram *N*_*a*_(*p*) starts to deviate down from the *p*^-*γ *^powerlaw behavior. This decline is an artifact of the inability of sequence-based algorithms such as BLAST to detect some of the bona fide paralogous pairs with low sequence identity. This explanation is corroborated by the observation that the exact position of the downturn of *N*_*a*_(*p*) in the region III is determined by the E-value cutoff (see Additional file 1).

### Birth-and-death model of the proteome evolution

In an attempt to interpret the empirical features of the PID distribution described above we propose a simple stochastic birth and death model of the proteome evolution. It consists of a sequence of random gene duplications, deletions, and changes in amino-acid sequences of proteins they encode. Several versions of such models were previously studied [4-7] most recently in the context of powerlaw distribution of family sizes. Our model extends these previous attempts by concentrating on evolution of sequence identities as opposed to just the number of proteins in different families.

**Figure 2:**
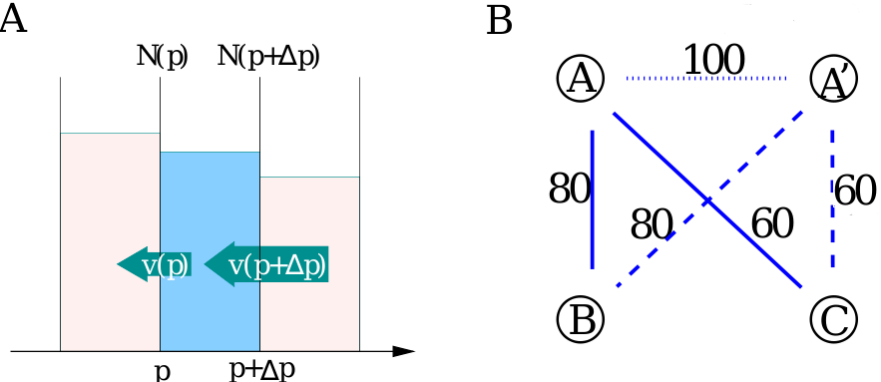
**Illustration of dynamical processes changing the PID histogram**. (panel A) The PID decay generates a negative flux *v*(*p*)*N*_*a*_(*p*) down the PID-axis. The net flux into a given bin Δ*p *is given by *v*(*p *+ Δ*p*)*N*_*a*_(*p *+ Δ*p*) - *v*(*p*)*N*_*a*_(*p*) ≈ $\Delta p\frac{d}{dp}[v(p)N_{a}(p)]$ (panel B) A single gene duplication event *A *→ *A' *gives rise to three new paralogous pairs: *A' *- *A*, *A' *- *B *and *A' *- *C*. Immediately after the duplication the pair *A *- *A' *has the PID = 100%, while PIDs of *A' *- *B *and *A' *- *C *are equal to those of *A *- *B *and *A *- *C*. Thus the PID of every previously existing paralogous pair involving *A *gets duplicated along with the duplication *A *→ *A'*.

Amino acid substitutions, insertions and deletions cause the sequence identity of any given pair of paralogous proteins to decay with time. Consider two paralogous proteins with PID = *p *× 100% aligned against each other. In the simplest possible case changes in their sequences happen uniformly at all amino acid positions at a constant rate *μ *= const. The effective "substitution" rate *μ *combines the effects of actual substitutions and short indels. The PID of this paralogous pair changes according to the equation *dp/dt *= -2*μp*. The factor two in the right hand side of this equation comes from the fact that substitutions can happen in any of the two proteins involved, while the factor *p *– from the observation that only changes in parts of the two sequences that remain identical at the time of the given change lead to a further decrease of the PID. This equation results in an exponentially decaying PID: *p*(*t*) *~ *exp(-2*μt*). More generally the drift of PID could be described by the equation *dp/dt *= -*v*(*p*). When substitution rate varies for different amino acids within the same protein the relationship between *v*(*p*) and *p *would in general be non-linear. For our immediate purposes we will leave it unspecified. The negative drift of PIDs generates a *p*-dependent flux of paralogous pairs down the PID axis given by *v*(*p*)*N*_*a*_(*p*). The net flux into the PID bin of the width Δ*p *centered around *p *is given by $\Delta p\frac{\partial }{\partial p}[v(p)N_{a}(p)]$ (see Fig. 2A).

Our model also involves random gene duplication and deletion events (birth and death of new protein-coding genes) which happen at rates *r*_dup _and *r*_del _correspondingly. In Fig. 2B we illustrate the details of how a gene duplication event creates new pairs of paralogous proteins. When a gene A is duplicated to A' a new pair of paralogs with PID = 100% is created (dotted line) and added to the rightmost bin of the PID histogram. Furthermore the freshly created gene A' inherits both paralogous partners (B and C) of the gene A. The PIDs of these two newly created paralogous pairs A'-B and A'-C (dashed lines) are also added to the respective bins in the histogram. Thus a duplication of any of the two paralogous genes with PID = *p *among other things results in the creation of a new pair of paralogs with the same PID. This process increases *N*_*a*_(*p*) at a rate 2*r*_dup_*N*_*a*_(*p*). Similarly the deletion of any of the two genes in this paralogous pair decreases *N*_*a*_(*p*) at the rate 2*r*_del_*N*_*a*_(*p*). The bin containing PID = 100% (*p *= 1) has an extra flux term *r*_dup_*N*_genes _from PIDs of the freshly created pair of duplicated genes (A-A' in our example). Here *N*_genes _is the total number of protein-coding genes in the genome. Adding up contributions of the three main processes (substitutions, duplications and deletions) one gets

$$\begin{array}{lll} \frac{\partial N_{a}(p,t)}{\partial t} & = & \frac{\partial }{\partial p}[v(p)N_{a}(p,t)]\\ & + & 2r_{\text{dup}}N_{a}(p,t)-2r_{\text{del}}N_{a}(p,t)+r_{\text{dup}}N_{\text{genes}}\delta (p-1). \end{array}$$

In our model the total number of genes *N*_genes _in the genome exponentially grows (or decays) according to *dN*_genes_/*dt *= (*r*_dup _- *r*_del_)*N*_genes_. When the genome size of an organism remains (approximately) constant with time one can find the stationary asymptotic solution of the previous equation. In this case one must have *r*_dup _= *r*_del _so that the second term in the right hand side is equal to zero.

In the case of an exponentially growing or shrinking genome the stationary solution for *N*_*a*_(*p*, *t*) does not exist. However, it exists for the histogram normalized by the total number of protein pairs: It is easy to show that since

∂*n*_*a*_(*p*, *t*)/∂*t *= (∂*N*_*a*_(*p*, *t*)/∂*t*)/[*N*_genes_(*N*_genes _- 1)/2] - 2(*r*_dup _- *r*_del_)*N*_*a*_(*p*, *t*)/[*N*_genes_(*N*_genes _- 1)/2] the equation for normalized PID histogram *n*_*a*_(*p*, *t*) acquires an extra negative term -2(*r*_dup _- *r*_del_)*n*_*a*_(*p*, *t*). This term exactly cancels the duplication and deletion terms in the equation for *N*_*a*_(*p*, *t*) and considerably simplifies the equation for the normalized histogram :

$$\frac{\partial n_{a}(p,t)}{\partial t}=\frac{\partial }{\partial p}[v(p)n_{a}(p,t)]+\frac{2r_{\text{dup}}}{N_{\text{genes}}-1}\delta (p-1).$$

In the steady state solution one has ∂*n*_*a*_(*p*, *t*)/∂*t *= 0 = ∂/∂*p*[*v*(*p*)*n*_*a*_(*p*)] or

*n*_*a*_(*p*) ~ *N*_*a*_(*p*) ~ 1/*v*(*p*).

The conjecture that the normalized PID histogram *n*_*a*_(*p*) = *N*_*a*_(*p*)/[*N*_genes_(*N*_genes _- 1)/2] indeed is nearly stationary during the course of evolution is corroborated by the fact that all six *n*_*a*_(*p*) curves in various genomes used in our study approximately lie on top of each other in Fig. 1B (compared to unnormalized *N*_*a*_(*p*) shown in Fig. 1A).

Comparing the Eq. 2 with the empirical form of *N*_*a*_(*p*) ~ 1/*p*^4 ^in the region II of Fig. 1 one concludes that the drift velocity in real genomes must obey *v*(*p*) ~ *p*^4^. Such a non-linear dependence of *v*(*p*) could be explained by the variability of the effective substitution rate within proteins (intra-protein variability). Assuming the intra-protein variability of substitution rates *μ *described by a PDF *ρ*(*μ*) one gets the following expression for *p*(*t*) and *v*(*t*):

$$p(t)=\int_{0}^{\infty }\rho (\mu )e^{-2\mu t}d\mu ;$$

$$v(t)=-\frac{dp(t)}{dt}=\int_{0}^{\infty }2\mu \rho (\mu )e^{-2\mu t}d\mu .$$

Eq. 3 is a generalization of the previously discussed exponential decay of *p*(*t*) derived for a constant substitution rate *μ*. It simply weighs these exponentials by *ρ*(*μ*) – their likelihood of occurrence. For any given *ρ*(*μ*) one could exclude time from Eqs. 3 and 4 and express *v *as a function of *p*. Such *v*(*p*) dependence could then be directly compared with the empirically derived formula. In the absence of an analytical expression relating *v*(*p*) to (*μ*) one is limited to use a trial-and-error method. We start with Gamma-distributed *ρ*(*μ*) ~ *μ*^*α*-1 ^exp(-*μ*/*μ*_0_) which has been predominantly used in the literature [8-10]. Inserting thus defined *ρ*(*μ*) into Eqs. 3 and 4 one gets *p*(*t*) = (2*μ*_0_)^-*α*^/(*t *+ (2*μ*_0_)^-1^)^*α *^and *v*(*t*) = *α*(2*μ*_0_)^-*α*^/(*t *+ (2*μ*_0_)^-1^)^*α*+1 ^which leads to *v*(*p*) ~ *p*^(*α*+1)/*α *^and thus to *N*_*a*_(*p*) ~ *p*^-(*α*+1)/*α*^.

### Robustness of the functional form of *N*_*a*_(*p*) with respect to assumptions used in the model

The birth-and-death model described above is based on a simplified picture of genome evolution. In particular it implicitly assumes:

• The neutrality of individual gene duplication and deletion events resulting in identical rates of these two processes in all paralogous families in the genome.

• Identical average amino-acid substitution rates *μ*_0 _in all individual proteins.

Both of these assumptions are known to be not, strictly speaking, true. Sequences of some "important" proteins (e.g. constituents of the ribosome) are known to evolve very slowly. Also, the families containing essential (lethal knockout) genes were recently shown [11] to be characterized by higher average duplication and deletion rates than those lacking such genes.

However, the validity of our main results goes well beyond the validity of the approximations that went into our birth-and-death model. The advantage of using the histogram of sequence identities generated by the all-to-all alignment (*N*_*a*_(*p*)) lies in its remarkable universality and robustness. When the Eq. 2 is applied to individual families one can see that family-to-family variation of (and correlations between) the duplication rate *r*_dup_, the deletion rate *r*_del_, and the average substitution rate *μ*_0 _affect only the prefactor in the powerlaw form of *N*_*a*_(*p*). Thus the exponent *γ *= 1 + 1/*α *describing this powerlaw is very robust with respect to assumptions of the model and depends only to the exponent *α *quantifying the *intra-protein *variability of amino-acid substitution rates.

The exact mechanisms behind this apparent universality of α are not entirely clear. Chances are that it is dictated more by the protein physics rather than by organism-specific evolutionary mechanisms. A possible path towards derivation of the exponent α from purely biophysical principles starts with the results of Ref. [12], which models the effects of (correlated) multiple amino-acid substitutions on stability of the native state of a protein. However, such analysis goes beyond the scope of the present work and will be reserved for a future study.

### Distribution of sequence identities of true duplicated pairs

A highly redundant dataset consisting of all paralogous pairs present in the genome enabled us to quantify the variability of intra-protein substitution rates. Another set of important parameters describing proteome evolution are average deletion/inactivation and duplication rates *r*_del _and *r*_dup_. As will be shown in the following, the reduced non-redundant dataset consisting only of protein pairs directly produced in duplication events allows us to estimate these rates.

To better understand the difference between those two datasets we illustrate it with a simple example. The family of four evolutionary related proteins A, B, C, D contributes six paralogous pairs to *N*_*a*_(*p*). This family was actually created by three subsequent duplication events: first A duplicated to give rise to B, then B duplicated to C and finally C duplicated to D. Thus only three out of total six paralogous pairs are directly produced in gene duplication events. The actual number of duplicated pairs could be even smaller if some intermediate genes were deleted in the course of the evolution. In general a family consisting of *F *proteins contributes at or around *F*(*F *- 1)/2 paralogous pairs to *N*_*a*_(*p*), but only *F *- 1 duplicated pairs to *N*_*d*_(*p*).

Nothing in the BLAST output for a given paralogous pair contains any information if it should or should not be included in the *N*_*d*_(*p*). However, using the set of all sequence identities of proteins for a given family one could tentatively reconstruct the course of duplication events that led to the appearance of this family. Generally speaking, this is a rather complicated task involving reconstructing the actual phylogenetic tree for every family in a genome. In this study we use a much simpler alternative based on the Minimum Spanning Tree (MST) algorithm (see Methods for more details). For each protein family this algorithm generates a tentative set of duplication events in its past history. Numbers of pairs included in *N*_*a*_(*p*) and *N*_*d*_(*p*) distributions in different organisms are listed in the Table 2.

**Table 2:** Statistics of datasets used in this study. The first column is the name of the organism, the second column – the number of protein-coding genes in its genome, *N*_genes_, the third column – the number of proteins for which we found at least one paralogous partner, the fourth column is the percentage of proteins with at least one paralog, the fifth column – the total number of distinct BLAST hits generated before we applied subsequent filtering, the sixth column – the number of paralogous pairs included in *N*_*a*_(*p*), and the seventh column – in *N*_*d*_**(***p*).

**Organism**	Proteome size	Number of proteins with paralogs	% of proteins with paralogs	BLASTP hits	Number of pairs in *N*_*a*_(*p*)	Number of pairs in *N*_*d*_(*p*)
*H. pylori*	1590	230	14%	3228	260	148
*E. coli*	4288	1428	33%	16768	2614	1013
*S. cerevisiae*	5885	1689	29%	43915	2297	1025
*C. elegans*	19099	6894	36%	204398	46463	5545
*D. melanogaster*	14015	4153	30%	557047	17621	3238
*H. sapiens*	25319	9252	37%	1330721	31078	6595

The dynamics of the distribution of duplicated pairs *N*_*d*_(*p*, *t*) is described by simply excluding the duplication term 2*r*_dup_*N *(*p*, *t*) from the equation for *N*_*a*_(*p*, *t*). Indeed, this term is caused by PIDs of non-duplicated paralogs A'-B and A'-C (dashed lines in Fig. 2(B)) generated when a gene A was duplicated. However, only the actual duplicated pair A-A' with initial PID of 100% (dotted line in Fig. 2(B)) is included in the distribution of duplicated pairs *N*_*d*_(*p*). Thus the dynamics of *N*_*d *_is described by

$$\frac{\partial N_{d}(p,t)}{\partial t}=\frac{\partial }{\partial p}[v(p)N_{d}(p,t)]-2r_{\text{del}}N_{d}(p,t)+r_{\text{dup}}N_{\text{genes}}\delta (p-1).$$

Once again the stationary solution exists for the normalized distribution. However, in this case the correct normalization factor is given by *N*_genes _and not *N*_genes_(*N*_genes _- 1)/2 as for *N*_*a*_(*p*). Indeed, every duplication event increasing the number of genes by one adds just one duplicated pair to *N*_*d*_(*p*) but up to *N*_genes _pairs to *N*_*a*_(*p*). Thus the normalized PID histogram of duplicated pairs *n*_*d*_(*p*, *t*) = *N*_*d*_(*p*, *t*)/*N*_genes _evolves according to

$$\frac{\partial n_{d}(p,t)}{\partial t}=\frac{\partial }{\partial p}[v(p)n_{d}(p,t)]-(r_{\text{dup}}+r_{\text{del}})n_{d}(p,t)+r_{\text{dup}}\delta (p-1).$$

According to our empirical findings the average rate of sequence divergence of paralogous proteins in most organisms is described *v*(*p*) = 2$\bar{\mu }$*p*^*γ*^, where $\bar{\mu }$ is the substitution rate averaged over all amino-acid positions in all proteins, and *γ *≈ 4 is the exponent related to the intra-protein variability of *μ*. The steady state of Eq. 5: ∂*n*_*d*_/∂*t *= 0 is satisfied by:

$$N_{d}(p)\sim n_{d}(p)=\frac{r_{\text{dup}}}{2\bar{\mu }p^{\gamma }}\exp\left(-\frac{r_{\text{dup}}+r_{\text{del}}}{2\bar{\mu }(\gamma -1)p^{\gamma -1}}\right).$$

### Numerical test of analytical predictions

**Figure 3:**
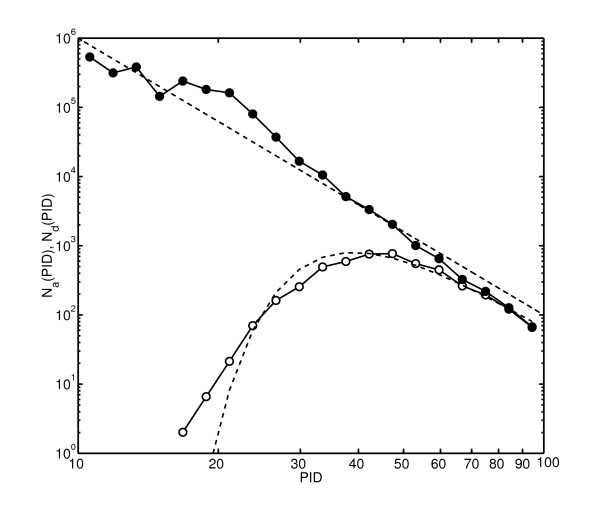
**Histogram of sequence identities in a numerical simulation of proteome dynamics**. The histogram of sequences identities of all paralogous pairs *N*_*a*_(*p*) (filled circles) and duplicated pairs *N*_*d*_(*p*) (open circles) in an artificial proteome generated by our numerical model for *α *= 0.33 and *r*_dup_/$\bar{\mu }$ = *r*_del_/$\bar{\mu }$ = 0.25 as described in the text. Solid line has the slope -4, while the dashed one is given by 6 with *γ *= 4 and the best fit value of *r*_del_/$\bar{\mu }$ = 0.237.

The analytical results derived above were confirmed by a numerical simulation. An artificial "proteome" used in our numerical model consists of a fixed number of "proteins" of identical lengths. At every timestep one takes the sequence of a randomly selected protein and uses it to overwrite the sequence of another randomly selected protein. This corresponds to a stationary genome case when *r*_dup _= *r*_del_. Each combined duplication/deletion event is followed by random substitutions of several "amino-acids" (see Methods for details of our simulation). In the beginning of the simulation each amino-acid position within every protein was randomly assigned a substitution rate drawn from the Gamma-distribution with *α *= 1/3. The proteome generated by this dynamical process is periodically analyzed in terms of sequence identity of all pairs of its proteins. The resulting distributions *N*_*a*_(*p*) (filled circles) and *N*_*d*_(*p*) (open circles) are shown in Fig. 3. They agree quite well with our theoretical predictions: *N*_*a*_(*p*) ~ 1/*p*^4 ^(solid line) and *N*_*d*_(*p*) ~ 1/*p*^4 ^exp(-*r*_del_/[3*μp*^3^]) (dashed line). The best fit to *N*_*d*_(*p*)/*N*_*a*_(*p*) generated in our numerical simulation with *A *exp(-*r*_del_/[3*μp*^3^]) gives *r*_del_/*μ *= 0.237 in excellent agreement with the actual value of *r*_del_/*μ *= 0.25 used in our simulation. This demonstrates that evolutionary parameters can be successfully reconstructed from the shapes of *N*_*d*_(*p*) and *N*_*a*_(*p*). This is especially encouraging in case of *N*_*d*_(*p*) because of the approximations that went into identifying true duplicated (sibling) pairs by the Minimum Spanning Tree algorithm.

### Fitting evolutionary parameters of real proteomes: the long-term deletion rate

To estimate the average of deletion and duplication rates we performed a two-parameter fit to the *N*_*d*_(*p*)/*N*_*a*_(*p*) ratio with *A *exp(-*B*/(*γ *- 1)*p*^*γ*-1^) (see Eqs. 2,6) in the 30% <*p *< 90% interval (region II in Fig. 1). Here *A *and *B *= (*r*_dup _+ *r*_del_)/(2$\bar{\mu }$) are the two free fitting parameters. The exponent γ used in the fitting formula itself was obtained from the best fit to *N*_*a*_(*p*) in the same region with the power-law form *p*^-*γ *^(see column 3 in Table 1). The ratio *r*_del_/$\bar{\mu }$ was extracted from the best-fit value of *B *and the independently calculated duplication rate ratio *r*_dup_/$\bar{\mu }$ (see subsection below). It is listed in the sixth column of the Table 1.

### Fitting evolutionary parameters of real proteomes: the short-term deletion rate of recent duplicates

A very pronounced and reproducible feature in all organism-wide histograms is an abrupt drop as is lowered from 100% down to about 90–95% (region I in Fig. 1.) The drop is as large as 30-fold in prokaryotes and is around 3-to-10 fold in eukaryotes. It is subsequently followed by a increase of in the region II which at low 25% (region III) turns down again only due to limitations of our ability to detect evolutionary related sequences. There exists several possible explanations for this initial drop in the region I :

• The gene conversion process. In a gene conversion process a part or the whole sequence of one of the paralogous genes is used as a template to modify the sequence of another. It happens with a reasonable frequency only if those two genes are sufficiently close to each other in their sequences so that DNA repair mechanisms might mistakenly assume that one of them is the corrupted version of the other. If gene conversion events are sufficiently common, the initial separation of a pair of freshly duplicated genes may take a long time, as one of them would be getting constantly converted back to the other. This would result in an abnormally small drift velocity *v*(*p*) for *p *close to 100% and hence to an abnormally high *N*_*a*_(*p*) ~ 1/*v*(*p*).

Another, more plausible explanation is that freshly duplicated genes are characterized by a much higher deletion rate $r_{\text{del}}^{*}$ ≫ *r*_del _[3]. Functional roles of such genes have not had enough time to diverge from each other making each of them more disposable than an average gene in the genome. Indeed, for *S. cerevisiae *and *C. elegans *it was empirically demonstrated [13,14] that the deletion or inactivation of genes with a highly similar paralogous partner in the genome is up to 4 times more likely to have no consequences for the survival of the organism than the deletion/inactivation of genes lacking such a partner.

The *N*_*a *_dynamics in the region I (*p *≃ 100%) is then described by

$$\frac{\partial N_{a}(p,t)}{\partial t}=\frac{\partial }{\partial p}[2\bar{\mu }N_{a}(p,t)]+(2r_{\text{dup}}-2r_{\text{del}}^{*})N_{a}(p,t)+r_{\text{dup}}N_{\text{genes}}\delta (p-1)$$

while the normalized distribution *n*_*a *_= *N*_*a*_/[*N*_genes_(*N*_genes _- 1)/2] obeys

$$\frac{\partial n_{a}(p,t)}{\partial t}=\frac{\partial }{\partial p}[2\bar{\mu }n_{a}(p,t)]+(2r_{\text{del}}-2r_{\text{del}}^{*})n_{a}(p,t)+\frac{2r_{\text{dup}}}{N_{\text{genes}}-1}\delta (p-1)$$

Here 2$\bar{\mu }$ = *v*(100%) is the average substitution rate in freshly duplicated pairs and $r_{\text{del}}^{*}$ is the deletion rate inside region I. The equation has an exponentially decaying stationary solution which for $r_{\text{del}}^{*}$ ≫ *r*_del _is simply given by *n*_*a*_(*p*) ~ exp($r_{\text{del}}^{*}p/\bar{\mu }$) This functional form is consistent with the empirical data for *p *just below 100% and the best fits to $r_{\text{del}}^{*}/\bar{\mu }$ are listed in the seventh column of the Table 1. Ref. [3] analyzed the distribution of silent substitution numbers per silent site *K*_*s *_between pairs of recently duplicated genes. Under the same "drift and deletion" hypothesis used to derive the Eq. 7 such *K*_*s*_-distribution *N*_*d*_(*K*_*s*_) should also have an exponential decaying form *Nd*(*K*_*s*_) ~ exp($-r_{\text{del}}^{*}K_{s}/{\bar{\mu }}_{s}$), where ${\bar{\mu }}_{s}$ is the average drift velocity of *K*_*s *_immediately following the duplication event. Fits to this exponential functional form performed in Ref. [3] resulted in $r_{\text{del}}^{*}/{\bar{\mu }}_{s}$ ~ 7 – 24. Our estimates $r_{\text{del}}^{*}/\bar{\mu }$ ~ 20 – 70 are consistent with those of [3] provided that the $\bar{\mu }/{\bar{\mu }}_{s}$*s *ratio is in 0.1 – 1 interval.

### Fitting evolutionary parameters of real proteomes: long- and short-term duplication rates

The number of paralogous pairs with PID≃100% also contains information about the raw duplication rate $r_{\text{dup}}^{*}$ in the genome. This rate is subsequently trimmed down to its long-term stationary value *r*_dup _by the removal of a large fraction of freshly created pairs as described in the previous subsection. New pairs with PID = 100% are created at a rate $r_{\text{dup}}^{*}$*N*_genes_, while they leave the bin containing PID = 100% at a rate 2$\bar{\mu }$*N*_*a*_(100%)/Δ*p*. Here Δ*p *is the width of the bin and *N*_*a*_(100%) is the number of pairs in this last bin. The width of the bin is assumed to be small enough so that the removal of genes from the bin due to deletion is negligible in comparison to that due to the drift in their sequences. Thus $r_{\text{dup}}^{*}/\bar{\mu }$ = 2*N*_*a*_(100%)/(*N*_genes_Δ*p*). The average duplication rates calculated this way are presented in the fourth column of Table 1. They are compatible with $r_{\text{dup}}^{*}/{\bar{\mu }}_{s}$ calculated in [15], where the same idea was applied to *N*_*d*_(*K*_*s*_).

The rate $r_{\text{dup}}^{*}$ includes the creation of some extra duplicated pairs which are then quickly (on an evolutionary timescale) eliminated from the genome during a "trial period" while their PID>90%. We have already demonstrated that such a deletion happens at a very high rate $r_{\text{del}}^{*}$ and thus has to be treated separately from the background deletion rate *r*_del_. The duplication rapidly followed by a deletion does not change the overall distribution of paralogous pairs. Therefore, the long-term average duplication rate *r*_dup _used in Eqs. 2, 6 is in fact considerably lower than the raw duplication rate $r_{\text{dup}}^{*}$. An approximate way to calculate it is to use power-law fits to *N*_*a*_(*p*) in the region II to extrapolate it up to 100%. Such extrapolated value $N_{a}^{\text{ext}}$(100%) could then be used to calculate the long-term average duplication rate as *r*_dup_/$\bar{\mu }$ = 2$N_{a}^{\text{ext}}$(100%)/(*N*_genes_Δ*p*). (see the fifth column of Table 1).

## Discussion and Conclusion

### An estimate of the number of superfamilies in different genomes

Any sequence-based method is bound to miss similarities between some of the distant paralogs. The situation could be somewhat improved [7] if one compares proteins' three-dimensional structures which are conserved over longer evolutionary times. The addition of previously undetected paralogous pairs results in some of the sequence-based families merging together to form larger superfamilies. Our empirical observations allow us to estimate the number of such superfamilies contained in a given genome. Indeed, the fraction of paralogous pairs among all gene pairs in a genome consisting of *N*_*F *_mutually unrelated superfamilies is given by 1/*N*_*F*_. A rough estimate of *N*_*F *_is provided by extrapolation of the *p*^-4 ^powerlaw into the Region III down to some cutoff PID_min_:

$$N_{F}=\frac{N_{\text{genes}}(N_{\text{genes}}-1)}{2}/\int_{{\text{PID}}_{\min}}^{1}Ap^{-4}dp.$$

Here A is the best fit to *N*_*a*_(*p*) with the *p*^-4 ^in the region II. Remarkably, the results of such calculation are roughly genome independent. Using the lowest theoretical limit PID_min _= 5% (the sequence identity of two unrelated sequences composed of 20 amino-acids) results in the effective number of superfamilies *N*_*F *_ranging between 4.7 in *C. elegans *and 9.9 in *D. melanogaster*. A more realistic limit PID_min _= 8% [7], which takes into account the non-uniform frequency among 20 amino-acids, somewhat increases the number of superfamilies to 36, 28, 31, 19, 40, and 35 for *H. pylori*, *E. coli*, *S. cerevisiae*, *C. elegans*, *D. melanogaster*, and *H. sapiens *correspondingly. These numbers are still respectably small compared to *N*_*F *_≃ 1000 one gets by using the cutoff PID_min _= 25% imposed by the inadequacy of sequence-based methods to detect similarity of remote paralogs.

### The exponent *α *in large individual families

The Gamma-distribution ~ *μ*^*α*-1 ^exp(-2*μ*/*μ*_0_) was traditionally used to model and fit the distribution of substitution rates in individual families of proteins (this tradition goes back to [16]). Our approach extends this approach to a proteome-wide scale and demonstrates that beyond its role as a *ad hoc *fitting function the Gamma-distribution indeed provides an excellent quantitative description of variability of intra-protein substitution rates.

The genome-wide value of the exponent *α *≃ 0.33 obtained in our analysis is consistent with its previous estimates in large protein families. For example, the best fits with the Gamma-distribution performed in Refs. [9,10] resulted in the exponent α in the 0.2 – 0.4 range. The authors of Ref. [8] quantified the variability of substitution rates using a large set of orthologous proteins in different genomes (this should be contrasted with paralogous proteins used in our analysis). The fits with Gamma-distribution resulted in a broad range of exponents α for individual proteins. Still the distribution of exponents α peaks around 0.25 (solid histograms in the Fig. 4 of Ref. [8]).

**Figure 4:**
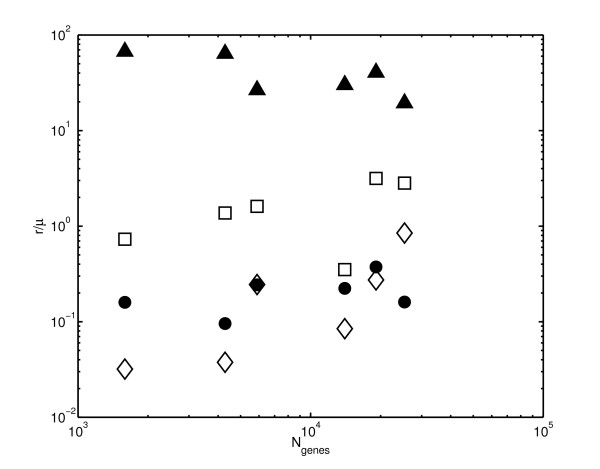
**Correlation between the number of genes in an organism and its duplication/deletion rates**. Evolutionary parameters *r*_dup_/$\bar{\mu }$ (open diamonds), *r*_del_/$\bar{\mu }$ (filled circles), $r_{\text{dup}}^{*}/\bar{\mu }$ (open squares), and $r_{\text{del}}^{*}/\bar{\mu }$ (filled triangles) plotted versus the total number of genes *N*_genes _in an organism. Organisms in the order of increasing number of genes are *H. pylori*, *E. coli*, *S. cerevisiae*, *D. melanogaster*, *C. elegans*, and *H. sapiens*. As explained in the text, more complex organisms (those with larger *N*_genes_) tend to be characterized by higher values of the first three ratios but lower values of the last ratio.

**Figure 5:**
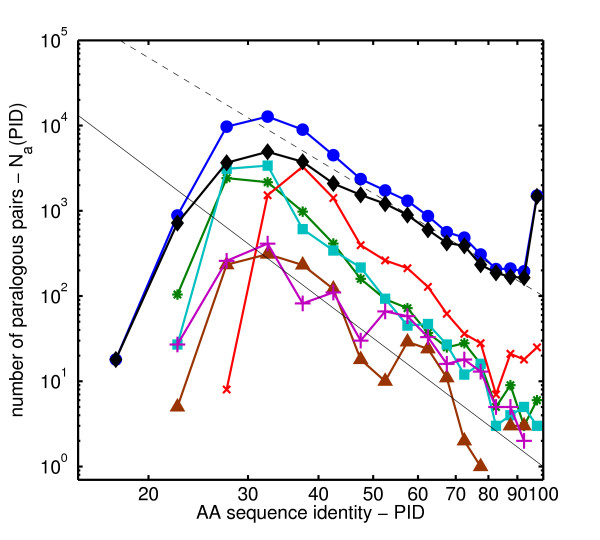
**Histogram of sequence identities of individual families in the genome of *C. elegans***. The histogram of amino acid sequence identities for pairs of paralogous proteins contained in each of the 5 largest families in the genome of *C. elegans*. Families in the order of decreasing size (measured by the number of proteins) are marked with green stars (243 proteins), cyan squares (188 proteins), red x's (162 proteins), brown triangles (105 proteins), and magenta +'s (73 proteins). Solid blue circles show the distribution of all paralogous pairs in the genome (as in Figure 1), while solid black diamonds – what is left after removing the above 5 largest families. Dashed line corresponds to a power law with the slope -4, while the solid one – the slope -5.

In principle, our methods could be also applied to large individual families of paralogous proteins. However, only a few of the largest families in any genome contain sufficient number of paralogous pairs (up to *F*(*F *- 1)/2 in a family of size *F*) to have a meaningful individual *N*_*a*_(*p*) histogram. Fig. 5 shows the results of such analysis in *C. elegans *with individual curves corresponding to *N*_*a*_(*p*) in the 5 largest families of paralogous proteins. Without exception all these histograms are well fitted by *Cp*^-*γ *^with *γ*_*LF *_≃ 5 – 6. This corresponds to the exponent *α *≃ 0.17 – 0.25, which is a somewhat lower than the exponent *α *= 0.33 that we observed for genomes as a whole.

Smaller individual families do not hold sufficient statistical power to analyze the shape of *N*_*a*_(*p*). One approach would be to group them together by some shared characteristic (e.g. by their size, or by whether or not they contain an essential gene as described in Ref. [11]). However, the *N*_*a*_(*p*) histogram in such a group would depend on additional parameters such as the rate of creation and removal of families of a given type and thus will not be amenable to our type of analysis. For example the collection of families binned by their size would have additional birth-and-death events due to whole families entering or leaving the selected bin. The rates of these processes would have a non-trivial dependence on the age of a family and thus cannot be easily incorporated into our mathematical framework.

It is important to emphasize once again that the exponent α quantifies only the *intra-protein *variability of substitution rates at different amino-acid positions within individual proteins. Such variability should not be confused with a much larger protein-to-protein variability of average substitutions rates. Indeed, sequences of different proteins encoded in the same genome are known to evolve at vastly different rates (see [8,10] and references therein). Some sequences, such as e.g. those of ribosomal proteins, remain virtually unchanged over billions of years of evolution, while others change at a much faster pace. In fact, the very importance of a protein is sometimes quantified by its average rate of evolution as more essential proteins involved in core cellular processes tend to evolve at slower than average rates.

### Genome size dependence and other properties of long- and short-term duplication and deletion rates

Our data indicate that the long-term duplication rate *r*_dup _is of the same order of magnitude as the long-term deletion rate *r*_del _(see columns 5 and 6 in the Table 1). This is to be expected since any large discrepancy in these rates would generate much greater differences in genome sizes than actually observed in these model organisms. However, as was proposed by [3], both of these rates are considerably smaller than their short-term ("raw") counterparts $r_{\text{dup}}^{*}$ and $r_{\text{del}}^{*}$ that include recently duplicated proteins.

Our results for the fruit fly *D. melanogaster *are consistent with an earlier observation [15] of an abnormally low average duplication rate in this organism. According to our data $r_{\text{dup}}^{*}/\bar{\mu }$ is about nine times lower than that in the genome of *C. elegans*. The long-term stationary duplication rate *r*_dup_/$\bar{\mu }$ in the fly is also the lowest in all eukaryotic genomes used in this study but is only three times lower than that in the worm.

Intriguingly, *r*_del_/$\bar{\mu }$, *r*_dup_/$\bar{\mu }$, and $r_{\text{dup}}^{*}/\bar{\mu }$ ratios are all positively correlated with the complexity of the organism quantified by the total number of genes in its genome (see correspondingly filled circles, open diamonds, and open squares in Fig. 4). This means that either the per-gene duplication rate in more complex organisms is consistently higher than in their simpler counterparts or that their average amino-acid substitution rate is lower. It is likely that both above trends operate simultaneously. One possible explanation for the latter trend is that the more sophisticated mechanisms of DNA copying and repair of higher organisms lead to lower average amino-acid substitution rates.

On the other hand, we find that the deletion rate of recent duplicates, $r_{\text{del}}^{*}/\bar{\mu }$, (filled triangles in Fig. 4) is negatively correlated with the number of genes in the genome. This result is in agreement with Ref. [17] where this trend was attributed to the decrease in effective population size in more complex organisms.

### The effects of whole genome duplications on the histogram of sequence identities

Two of the organisms used in our study (*S. cerevisiae *and *H. sapiens*) are characterized by a dramatically lower value of the power-law exponent *γ *(1.8 for yeast and 2.4 for human) and the overall poor quality of the power law fit to *N*_*a*_(*p*). One plausible explanation of this anomaly is in terms of Whole Genome Duplications (WGD) in lineages leading to these genomes. It is well established [18] that baker's yeast underwent a WGD event, which most likely occurred about 100 Myrs ago. While the subject remains controversial, it is also commonly believed that the vertebrate lineage leading to *H. sapiens *(among many other vertebrate genomes) also underwent one or several large-scale duplication events [19,20]. In the immediate aftermath of a WGD event the PID distribution changes as follows: *N*_*a*_(*p*) → 4*N*_*a*_(*p*) for *p *< 100%, while *N*_*a*_(100%) → 4*N*_*a*_(100%) + *N*_genes_. Indeed, every ancestral paralogous pair A-B would give rise to 3 new pairs with the same PID: A-B', A'-B, and A'-B'. At the same time the bin containing the PID = 100% would in addition get *N*_genes _(or fewer for a large segmental duplication) of freshly created duplicated pairs of the type A-A' and B-B'. The subsequent spread of this sharp peak at PID = 100% towards lower values of PID accompanied by a rapid deletion of redundant copies of duplicated genes would result in an effective flattening of the *N*_*a*_(*p*) histogram in its upper range and thus in lower effective value of the exponent *γ*.

**Figure 6:**
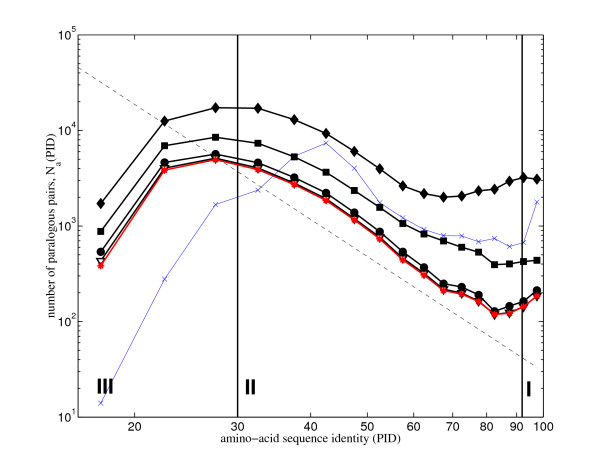
**Histogram *N*_*a*_**( ***p*) of sequence identities and four rounds Whole Genome Duplications (WGD) in *Paramecium tetraurelia***. The histogram of sequence identities of 103,828 paralogous pairs among 39,642 proteins in the genome of *Paramecium tetraurelia *(solid diamonds) detected by an all-to-all BLASTp alignment (see methods for details). Other histograms shown in this plot correspond to a progressive removal of new proteins created in the four WGD events [21]: 41,890 pairs among 27,616 proteins excluding those generated in the latest WGD event (solid squares), 25,342 pairs among 23,618 proteins excluding those generated in the last two WGD events (solid circles), 22,287 pairs excluding those generated in the latest three WGD events (open triangles), and 21,417 pairs among 22,635 proteins excluding those generated in all four known WGD events (red stars). For comparison we copy from Fig. 1A the histogram of 31,078 pairs among 25,319 *H. sapiens *proteins (blue *times*-es). One can see that by progressive elimination of pairs generated in WGD events the functional form of the *N*_*a*_(*p*) histogram in *Paramecium tetraurelia *approaches the universal scaling form: *N*_*a*_(*p*) ~ *p*^-4 ^(dashed line).

To further test this hypothesis we analyzed the recently sequenced genome [21] of a ciliate *Paramecium tetraurelia*. This organism underwent as many as four separately identifiable WGD events [21]. We used our standard methods to construct the PID histogram *N*_*a*_(*p*) from the all-to-all alignment of its nearly 40,000 genes. Due to the sheer size of this proteome we employed the same conservative 10^-30 ^E-value cutoff we used for *H. sapiens *and *C. elegans*. Solid diamonds in the Fig. 6 correspond to the full PID histogram in *Paramecium tetraurelia *consisting of all 103,828 paralogous pairs detected by our methods. Authors of Ref. [21] identified the lists of putative pairs of duplicated genes generated in each of the four WGD events in the lineage leading to this genome. By dropping one randomly-selected gene from these WGD pairs we generated the set of four progressively more narrow PID histograms. These histograms are also shown in Fig. 6: 41,890 pairs excluding the genes generated in the latest WGD event (solid squares), 25,342 pairs excluding the genes generated in the last two WGD events (solid circles), 22287 pairs excluding the genes generated in the latest three WGD events (open triangles), and 21,417 pairs excluding the genes generated in all four WGD events (red stars). For comparison, the Fig. 6 also reproduces the histogram of 31,078 pairs in the *N*_*a*_(*p*) of *H. sapiens *(blue ×-es). One can see that progressive elimination of pairs generated in WGD events gives rise to the *N*_*a*_(*p*) histogram approaching the universal scaling form: *N*_*a*_(*p*) ~ *p*^-4 ^(the dashed line in Fig. 6). Furthermore, the PID distribution of gene pairs generated in each of the WGD events has a shape that is qualitatively consistent with the predictions of our birth-and-death model. In particular, the gene pairs from the latest round of WGD did not have time to sufficiently diverge. As a result, their PID-distribution (shown in black in the Fig. 3a of the Ref. [21]) has a peak around 95% sequence identity with a half-maximum at 75%. The analysis of *Paramecium tetraurelia *genome provides an additional strong support to our conjecture that the unusually flat PID histograms in human and baker's yeast are caused by WGD events in lineages leading to these two organims.

## Methods

### The details of generating lists of paralogous proteins

The proteomes of *H. pylori *strain 26695 and *E. coli *strain K12-MG1655 were downloaded from the Comprehensive Microbial Resource (CMR) [22] version 1.0. Sequences of S. cerevisiae proteins are from the Saccharomyces Genome Database (SGD) [23] version number 20031001. The *D. melanogaster*'s sequences are from the Berkeley Drosophila Genome Project [24], release 3.1. *C. elegans *– Wormbase [25], release WS127.*H. sapiens *– the NCBI database [26], build 34.1. The initial set of paralogous pairs for each of the organisms was identified by an all-to-all alignment of sequences of its proteins to each other using the BLASTP program [1]. For *H. pylori*, *E. coli*, *S. cerevisiae*, and *D. melanogaster *genomes, the E-value threshold of 10^-10 ^was employed. This corresponds to p-values of the order of 10^-12 ^(for *H. pylori*) and lower. Due to larger genome sizes of *C. elegans *and *H. sapiens *an even more conservative E-value of 10^-30 ^was used to reduce the number of hits generated by the algorithm.

The "raw" datasets for worm, fly and human often contain multiple overlapping protein sequences predicted by different gene models of the same gene (including but not limited to different splicing variants). To avoid spurious hits we first mapped entries in raw datasets to unique gene IDs. This was easy to accomplish in the fly and worm datasets, where names of different gene models differ from each other by the last letter. In human genome, this was done by mapping the gi numbers of sequences in the raw dataset to unique GeneID (LocusLink) identifiers from the Entrez Gene database [27]. Subsequently, if multiple BLAST hits were connecting the same pair of gene IDs we kept the one with the longest aligned region. This way we were guaranteed that one and only one pair of splicing (or gene model) variants per pair of gene IDs would contribute to the PID histogram.

In all genomes, only pairs in which the aligned region constituted at least 80% of the length of the longer protein were kept [15]. This excludes contribution from pairs of multi-domain proteins paralogous over only one of their domains.

Initially, the PID histogram in *S. cerevisiae *had two very sharp peaks at 51% and 70%. A close inspection revealed that these peaks are produced by evolutionary related subfamilies of nearly identical transposable elements. To correct for this obvious artifact in *S. cerevisiae *we removed 108 proteins encoded by known transposable elements listed in the Saccharomyces Genome Database [23] and their homologs.

The overall shape of the PID histogram in regions I and II is not sensitive to the E-value cutoff chosen. In Fig. 1S we show that when the E-value cutoff in the fly dataset was changed from a less conservative 10^-10 ^to a more conservative 10^-30 ^value, the shape of the histogram above 40% remained virtually unchanged. Similarly, the results are nearly independent on the type of the BLOSUM substitution matrix used (in the end we opted for the BLOSUM45.) Finally, we verified that our results are independent of the alignment algorithm utilized to calculate PIDs. Indeed, in the fly dataset we have recalculated PIDs for all paralogous pairs detected by BLAST using much more sophisticated Smith-Waterman algorithm [28]. The resulting histogram (shown as blue stars in Fig. 1S) is virtually indistinguishable from that based on the blastp output.

### Numerical model of the proteome evolution

We numerically simulated a birth and death model mimicking the evolution of a fixed-size proteome by duplication, deletion and substitutions. We first randomly fill a 2,000 × 100 matrix with integer numbers ranging from 1 and 20 (20 types of "amino-acids"). This constitutes the initial state of our artificial genome/proteome, encoding 2,000 "proteins" of 100 "amino-acids" each. Every amino-acid position in each of the proteins is randomly assigned the substitution rate *μ *drawn from a Gamma-distribution with *α *= 1/3. One evolutionary timestep consists of:

1. Duplicate a randomly selected gene in the genome and use this duplicated copy to replace another randomly selected gene (deletion). Thus in this model the deletion rate is exactly equal to the duplication rate.

2. Randomly pick 400 amino-acid positions in the whole genome and substitute amino-acids at those positions to a randomly selected new value. The probability of a particular amino-acid position to be picked is proportional to its substitution rate *μ*.

This choice of parameters in our model corresponds to $r_{del}/\bar{\mu }=r_{del}^{*}/\bar{\mu }=r_{dup}/\bar{\mu }=r_{dup}^{*}/\bar{\mu }=0.25$. Indeed, the average substitution rate per amino acid during one timestep is given by 400/(100 × 2000) = 1/500. It is equal to 0.25 of the per-gene per timestep duplication/deletion rate of 1/2000. In this artificial evolutionary process we have the advantage of keeping track of all the duplicated pairs. Thus, after each duplication event the list of all duplicated pairs is updated and can be directly read off. After repeating the above steps for 20,000 times the full genome alignment of all proteins is produced and stored. The distributions of duplicated and all paralogous pairs shown in Fig. 3 are generated by averaging over 20 such samples.

### Identification of true duplicated (sibling) pairs by the Minimum Spanning Tree algorithm

We are naturally not in possession of the set of protein pairs that actually underwent duplication in the course of evolution of a given genome. The identification of the most likely candidates for these "true" duplicates is in general a rather complicated task which involves reconstructing the actual phylogenetic tree for every family in a genome. However, we could make a much simpler educated guess about past duplication events by connecting paralogous proteins in a given family with the Minimum Spanning Tree (MST) that is the tree maximizing the sum of PIDs along its edges (or, to agree with its name, minimizing its opposite sign value). For a family consisting of *F *proteins such tree has exactly *F *- 1 edges representing our best guess about the actual duplication events. One can prove the truth of this by induction: when a freshly duplicated pair is created with PID = 100% it extends the previously existing Minimum Spanning Tree of a family by one edge. Assuming a constant rate of divergence for all paralogous pairs in a given family, the set of duplicated pairs would continue to form the Minimum Spanning Tree at all times. We used the Kruskal algorithm [29] to approximately detect the MST.

### Detection of families of paralogous genes

Families of paralogous proteins used in Figure 5 are defined as mutually isolated clusters of proteins in the network in which paralogous pairs are connected by a link. Every two nodes within a family are either directly or indirectly connected to each other by at least one chain of paralogous links, while different clusters (families) are completely disconnected from each other. Because of our requirement for the length of the aligned region to be >80% of the length of the longest protein in a pair, all proteins within such families are rather homogeneous in their lengths.

## Authors' contributions

SM and designed the study and its analytical framework. KKY acquired the genomic data and performed the sequence alignment. SM and KKY analyzed the results. JBA and SM wrote the manuscript. JBA performed numerical simulations of the model proteome and the MST algorithm. All authors read and approved the manuscript.

## Reviewers comments

### Reviewer 1: Eugene V Koonin, National Center for Biotechnology Information, National Institute of Health, Bethesda, Maryland, USA

This is quite an interesting, elegant study that presents a mathematical model connecting the distribution of percent sequence identity in paralogous protein families with the parameter of the gamma-distribution of intra-protein variability. The latter parameter had been explored before, and the values reported here are within the previously estimated ranges, but to my knowledge, this is the first work that derives this parameter theoretically from completely independent data. It is intriguing and, I suppose, important that the distributions of the identities between paralogs and, accordingly, the gamma-distribution parameter are almost genome-independent. It seems like the latter parameter is almost a "fundamental constant" that follows from the physics of protein structure that is, of course, universal.

I have three comments that are rather technical but bear on the robustness and generality of the conclusions.

1. A trivial point ... but, I feel it would have been helpful to increase the number of analyzed genomes, both in terms of diversity, and by including more than one genome from each of the included lineages (and others). The analysis of sets of related genomes would (hopefully) demonstrate the robustness of the obtained distributions, and would also help assessing the significance of the differences in the exponents seen among genomes. In particular, similar, flat distributions found in human and in yeast are somewhat strange given the huge difference in the size and complexity of these genomes. This is attributed to the legacy of whole-genome duplications but I find that explanation dubious. Traces of this duplication in yeast and, especially, in vertebrates are very weak. Including more genomes would help to clarify this issue. The reported genome analysis is very simple, it cannot be computationally prohibitive.

#### Authors response

*We agree that extending our analysis to include more genomes is fairly straightforward. However, we want to save the subject of lineage-dependence of the exponent γ (apart from that related to Whole Genome Duplications (WGD)) for future studies and to report it in a separate publication. To check our hypothesis that WGD are responsible for unusual profile of the PID-histograms in human and yeast we analyzed the genome of Paramecium tetraurelia. The lineage leading to this organism underwent as many as four separately identifiable WGD events. The results of our analysis presented in Fig. *6* and the accompanying section of the manuscript have beautifully confirmed our initial hypothesis: while the all-to-all N*_*a*_(*p*) *histogram in the whole proteome of Paramecium tetraurelia (solid diamonds in Fig. *6*) has an unusually flat profile similar to the one we saw in human (blue ×-es in Fig. *6*), the removal of proteins generated in WGD events results in a stepper PID-histogram (red stars in Fig. *6*) which is in excellent agreement with the universal p*^-4 ^*functional form (dashed line in Fig. *6*). This provides necessary support to our original conjecture that unusually flat PID histograms in human and baker's yeast are also due to (possibly less obvious) duplicated pairs of proteins generated in WGD events in these two lineages.*

2. The mathematical model developed in the paper is a typical birth-and-death model. I wonder why the phrase is not used (it would immediately clarify the matter to those familiar with the field) and some of the relevant literature is not cited.

#### Authors response

*We have modified our notation to incorporate this comment. We also cited the appropriate literature on birth-and-death models *[4-6].

3. The "true" duplicates are identified using minimum spanning tree under the constant rate assumption. This is quite a crude method and an unrealistic assumption, too. Building actual phylogenetic trees, certainly, would be more appropriate. This might be too hard technically for this amount of material but, at least, the issues should be acknowledged, I think.

#### Authors response

In the Background section of the manuscript we now explicitly mention that the minimum spanning tree is just a simpler (yet less precise) alternative to reconstructing the actual phylogenetic tree for every family in a genome.

### Reviewer 2: Yuri Wolf, National Center for Biotechnology Information, National Institute of Health, Bethesda, Maryland, USA (nominated by Eugene Koonin)

The authors present an elegant model of protein evolution that ties together duplication, loss of paralogs and sequence divergence. Under the assumption of the gamma-distributed variation of intra-protein evolution rates the model correctly predicts the power-law shape of the distribution of distances between paralogs in fully sequenced genomes. Analysis of the observed distributions allows to estimate the long-term rates of duplication, retention of paralogs and the shape parameter of the intra-protein evolution rate distribution in different organisms.

The authors are very explicit and thorough about the model description. However one point should be emphasized for the sake of biologists among the Biology Direct readership: the model is based on neutral evolution of both protein sequence and the complement of paralogs in the family and assumes that all protein families behave in the same manner. This is, obviously, a gross (if necessary) simplification of reality. The good agreement between the model predictions and the observed data is quite amazing and possibly deserves some discussion.

#### Authors response

*I believe by this comment Dr. Wolf has raised an important point which was inadequately presented in our manuscript. While the model itself indeed was built using a simplified (completely neutral) picture of real evolutionary processes the results of this analysis turned out to be independent of these assumptions. Thus they are expected to remain valid in a more realistic evolutionary scenario (such as variable average substitution, gene duplication and deletion rates in individual families) when these assumptions are relaxed. We have added a new subsection "Robustness of the functional form of N*_*a*_(*p*) *with respect to neutrality assumptions" to the Results section of the manuscript which describes in details our answer to this comment.*

Probably the most interesting observation in the paper is the near-constancy of the power of the middle part of the distribution curve (~4) and, according to the model, the shape parameter of the intra-protein evolution rate distribution (~1/3). This result is in surprising agreement with earlier estimates, including our own [Grishin et al. 2000], obtained using entirely different approaches. This might be telling us that this parameter is a "universal constant" of protein evolution and that it is dictated more by protein physics rather than organism-specific properties.

#### Authors response

This is now also discussed in the new section mentioned in our previous response.

The section on the numerical simulations is somewhat less justified in the eyes of this reviewer. The underlying mathematical model appears to be fully solved analytically and simulations follow the model precisely. Thus the results of the simulations are expected to agree with the analytical solution unless some really dumb mistake was made in the course of derivation or in the implementation of the simulations. If I am missing something and the impact of this section goes beyond the simple verification, it probably should be discussed in the text.

#### Authors response

*We agree that for the most part we use numerical simulations just to confirm the validity of our analytical results. Still, we decided to keep it in the manuscript since it clearly demonstrates (see Fig. *3*) that the deletion rate used in the model could be successfully reconstructed from the N*_*d*_(*p*) *histogram. This is not entirely obvious because of the approximations that went into deducing the true duplicated (sibling) pairs using the Minimum Spanning Tree algorithm.*

### Reviewer 3: David Krakauer, Santa Fe Institute, Santa Fe, New Mexico, USA

The paper introduces a number of new ideas, including a minimal spanning tree algorithm for eliminating redundant distance information in a full pairwise distance matrix in order to yield an estimate of the true number of paralogous genes.

I tend to view this paper as a contribution to the neutrality literature which seeks to explain large scale patterns of genomic evolution in terms of fundamental mutational processes without invoking dedicated selection pressures acting on specific genes. Having said this, selection could be playing an important role in accounting for effective rate variation in amino acid substitutions, and in establishing the parameters of duplication and deletion that prevent excessive growth or shrinking of genomes. Whatever these selection pressures might be, they would seem to have to apply across a number of species.

I have a number of questions relating to the means of establishing the empirical power law, and the interpretation of the results.

1. As the authors are aware straight lines in log-log plots are not equivalent to having demonstrated a power law distribution and least squares fitting frequently generate biased estimates. Recent research (Clauset et al 2007) presents maximum likelihood estimators for scaling parameters free from these biases. How do the authors establish confidence in their estimates of the exponent?

#### Authors response

*Due to an unavoidably narrow range of our power law fits along the x-axis (The region II includes *0.3 <*p *< 0.95 *or half a decade) we didn't use any sophisticated techniques in our fitting protocols. In fact, in this interval the exponential fits look only marginally worse than those with a power law. Thus for us the exponent γ of a power law fit is just a convenient single parameter quantifying the distribution which is A) consistent with the Gamma-distribution traditionally used to describe substitution rates; B) nearly universal in a broad variety of organisms ranging from H. pylori to D. melanogaster.*

2. I was somewhat confused by the renormalization procedure for the raw distance histograms. While I understand that this is required in order to ensure a stationary distribution, I do not see clearly what the biological implications or assumptions of this step are. Perhaps this could be clarified?

#### Authors response

*A standard mathematical approach to describing stationary probability distributions in growing systems is to normalize the histogram in question by the sum of its elements (*∑_*p*_*N*_*a*_(*p*) *in our case). However, we noticed that the total number of paralogous pairs *∑_*p*_*N*_*a*_(*p*) *detected by all-to-all alignment of all protein sequences grows at the same exponential rate *2(*r*_dup _- *r*_del_) *as the square of the number of genes *- $N_{\text{genes}}^{2}$. *Moreover, in real genomes the relationship *∑_*p*_*N*_*a*_(*p*) ~ $N_{\text{genes}}^{2}$*holds very well (see Additional file *2*). Thus we decided to use the total number of gene pairs N*_genes_(*N*_genes _- 1)/2 ~ $N_{\text{genes}}^{2}$*(the theoretical upper bound to the number of paralogous pairs) to normalize the N*_*a*_(*p*)*. The biological implication of this result is that the fraction of paralogous pairs among all gene pairs is roughly the same in all genomes (as manifested by the collapse of normalized distributions in Fig. *1B*).*

*We have also added the new subsection "An estimate of the number of superfamilies in different genomes" in which we speculate that the p*^-4 ^*could be extended well into the region III down to its theoretical minimum of *5 – 8%*. This could be (at least partially) accomplished if in addition to sequence similarity one would use structural similarity to define superfamilies of paralogous proteins. These results indicate that normalizing N*_*a*_(*p*) *by N*_genes_(*N*_genes _- 1)/2 *is a remarkably close approximation to normalizing it by the actual (presently unknown) number of paralogous pairs contained in the genome (including the evolutionary relationships missed by sequence-alignment algorithms).*

3. I worried a little about the uniqueness of the Gamma-distribution in generating the scaling behavior given the absence of a robust test for the scaling exponent. How many different distributions have been tested, and how robust is the result to departure from the Gamma?

#### Authors response

*As we explained in our response to your question #1, due to an inherently narrow range of the Region II (half a decade) we never state that the power law functional form resulting from Gamma-distributed substitution rates μ is the unique way to mathematically describe the N*_*a*_(*p*) *histogram in real genomes. Thus we didn't test multiple μ-distributions. Our only claim is that the observed shape of N*_*a*_(*p*) *is consistent with Gamma-distributed (α *= 0.33*) substitution rates of individual amino-acids within a protein.*

4. A little more could have gone into the discussion on the mutational processes and the role of selection. Should we assume rate variation to be the outcome of selection (as in the example of the slow rate of evolution of ribosomal proteins) where perhaps an active site remains more highly conserved, or is it the contention of the authors that some purely stochastic process at the mutational level accounts for this variation? A similar argument could be made for the duplication and deletion equilibrium. As the paper reads now, I am not sure what processes the authors have in mind.

#### Authors response

*These and other points are now explained in the new subsection "Robustness of the functional form of N*_*a*_(*p*) *with respect to assumptions used in the model" added to the Results section of the manuscript.*

5. Surely the study of evolution through the properties of genetic distance histograms is older than Lynch and Conery (2000). I am aware of earlier work by Gillespie and many others.

#### Authors response

Thank you for pointing this out. We have added the book by Gillespie and references therein to our citation list.

6. The paper needs to be spell checked and grammar checked.

#### Authors response

Done.

### Reviewer 4: Eugene Shakhnovich, Harvard University, Cambridge, Massachusetts, USA

Power-law distributions are ubiquitous in Protein Universe and were reported to describe distribution of sizes of gene families, fold families, structural similarity relationships and other properties (1–4). It had been widely accepted that the underlying reason for their emergence is in evolutionary dynamics of creation of new genes and proteins and several dynamics models have been proposed to describe it (1, 3, 5, 6). Here the authors study the distribution of amino acid sequence similarities in paralogous families and also find a regime where power-law describes the observed histogram well. They proposed a mathematical model akin to master equation approach which essentially assumes that the rate of divergence is non-uniform it depends on the sequence ID of genes in question. The model and the analysis are interesting and make a valuable contribution to the literature on evolutionary dynamics. The major strength of this study is in its highly quantitative character which provides interesting insights about duplication/deletion rates which are apparently dependent on past history. However I would like the authors to address the following questions:

1) The authors consider all gene families in various organisms regardless of their functional distributions. However recent work of B. Shakhnovich and Koonin (7) (which is in a sense a forerunner of the present paper) has demonstrated that evolution of paralogous families is dramatically different in the case of families containing essential (E-families) genes and families that do not contain such genes (N-families). It would be interesting to carry out the same quantitative analysis separately for E- and N-families and check how different exponents of intermediate power-law regimes are and how does it fit into ID-dependent divergence rate picture.

#### Authors response

*We agree that it would be interesting to separately analyze the PID-histogram E- and N-families. The Fig *3B* of the B. Shakhnovich and E. Koonin article (Ref. *[7]* in the list below) essentially does that. From it one can see that while E-families, which are on average larger (*[7]*) than N-families, have a sequence identity histogram similar to the whole-genome N*_*a*_(*p*) *in our study, the composite PID histogram of all N-families in yeast is essentially flat. However, as we now explain in the revised version of our manuscript our birth-and-death model does not apply to collections of individual families grouped together by some shared characteristic (e.g by their size or by whether or not they contain an essential gene). Indeed, the dynamics of such a group would depend on additional parameters such as the rate of creation and removal of families of a given type. For example, the appearance of an essential gene gene in an N-family would turn it into a new E-family and remove its contribution to the histogram of all N-families. We feel that modification of our analysis to incorporate these extra terms goes beyond the scope of this article.*

2) The major weakness of this analysis (and other phenomenological approaches) is that the "explanation" for power-law regime comes from an assumption of a certain form of the distribution of substitution rates in the form of Gamma-function. While assuming Gamma-function may result in good fits it is entirely mysterious why does it emerge. The authors make a very interesting hint that Gamma emerges from intra-protein variability of substitution rates but they do not dwell much further on that. In fact such variability does exist. It was quantitatively studied in a microscopic protein evolution model by Dokholyan and myself in 2001 [8]. It would be highly instructive to check whether distributions of substitution rates observed in [8] can provide additional insights into the microscopic origin of empirical fits used in this work.

#### Authors response

*It would be indeed extremely exciting to find a truly "microscopic" explanation of the universal parameters of the Gamma-distribution reported in our manuscript along the lines of the Ref. (*[8]*). We feel however, that this goes well beyond the scope of this article. In the new subsection of our manuscript "Robustness of the functional form of N*_*a*_(*p*) *with respect to assumptions used in the model" we now cite the Ref. (*[8]*) and mention that its results lead to a biophysical explanation to the remarkable universality of the exponent a reported in our manuscript.*

3) The effective distribution of intra-protein variability seems to depend on family size. Why? Can it be related to functional constrains (e.g. E- and N- families). A comment or further analysis will be helpful.

#### Authors response

*Soon after sending our manuscript for review we realized that our mathematical model is applicable only to whole genomes or large individual families and does not describe PID histograms in collections of many families grouped by their size. Indeed, such collections would have additional birth-and-death events due to whole families entering or leaving the selected bin of family sizes. The rates of these processes would have a non-trivial dependence on the age of a family and thus cannot be easily incorporated into our mathematical framework. Thus we removed the Fig. *5B* showing the apparent systematic variation of the exponent γ with the family size, which, in the hindsight, is likely caused by these extra birth-and-death terms. For the discussion of E- and N-families see our response to your question 2).*

4) The power-law regime is observed only at sufficient level of divergence. Why? How can current model be modified to account for the full histogram, not only its power-law part?

#### Authors response

*We attribute the upward turn in N*_*a*_(*p*) *for p *> 90% *(inside the Region I) to much higher deletion rates of recently duplicated genes caused by their apparent redundancy. The crossover to power law in the Region II means that this redundancy tends to be lost below this level of sequence identity. The combination of our equations (1) and (7) provide a comprehensive mathematical description valid in both Regions II and I (as we explained the crossover in the region III is an artifact caused by some bona fide paralogous pairs being missed by sequence alignment algorithms).*

**References used by Eugene Shakhnovich: **1. Qian, J., Luscombe, N. M. & Gerstein, M. (2001) J Mol Biol 313, 673–81.

2. Koonin, E. V., Wolf, Y. I. & Karev, G. P. (2002) Nature 420, 218–23.

3. Huynen, M. A. & van Nimwegen, E. (1998) Mol Biol Evol 15, 583–9.

4. Dokholyan, N. V., Shakhnovich, B. & Shakhnovich, E. I. (2002) Proc Natl Acad Sci U S A 99, 14132–6.

5. Karev, G. P., Wolf, Y. I., Rzhetsky, A. Y., Berezovskaya, F. S. & Koonin, E. V. (2002) BMC Evol Biol 2, 18.

6. Roland, C. B. & Shakhnovich, E. I. (2007) Biophys J 92, 701–16.

7. Shakhnovich, B. E. & Koonin, E. V. (2006) Genome Res 16, 1529–36.

8. Dokholyan, N. V. & Shakhnovich, E. I. (2001) J Mol Biol 312, 289–307.

## Supplementary Material

Additional file 1 The overall shape of the PID histogram is independent of the alignment algorithm and the E-value cutoff. The PID histogram *N*_*a*_(*p*) in the fly (*D. melanogaster *genomes when pairs of paralogous proteins were detected using the blastp algorithm [1] with E-value cutoff of 10^-10 ^(filled circles) and 10^-30 ^(open diamonds). The inset shows the ratio of these two histograms, which is very close to 1 for *p *> 40%. Thus the overall shape of *N*_*a*_(*p*) in most of the Region II (Fig. 1) is nearly + cutoff independent. The *N*_*a*_(*p*) also is insensitive to a particular algorithm used to align the pairs. Indeed, when paralogous pairs detected by the blastp with the E-value cutoff of 10^-10 ^(filled circles) were realigned using the Smith-Waterman algorithm [28] the resulting distribution (blue stars) changed very little. Click here for file

Additional file 2 The quadratic scaling of the total number of paralogous pairs with the number of genes in the genome. The total number of paralogous pairs ∑_*p*_*N*_*a*_(*p*) generated by the all-to-all alignment of all protein sequences encoded in the genome (the y-axis) scales as the square of the total number *N*_genes _of protein-coding genes in the genome. Solid symbols are six model organisms used in our study. The solid line has the slope 2 on this log-log plot. Click here for file
