# Supplementary figures and images for: Parameters of proteome evolution from histograms of amino-acid sequence identities of paralogous proteins
Source: Biol Direct. 2007 Nov 26;2:32. doi: 10.1186/1745-6150-2-32 (PMC2246104; doi:10.1186/1745-6150-2-32)

Figure S1

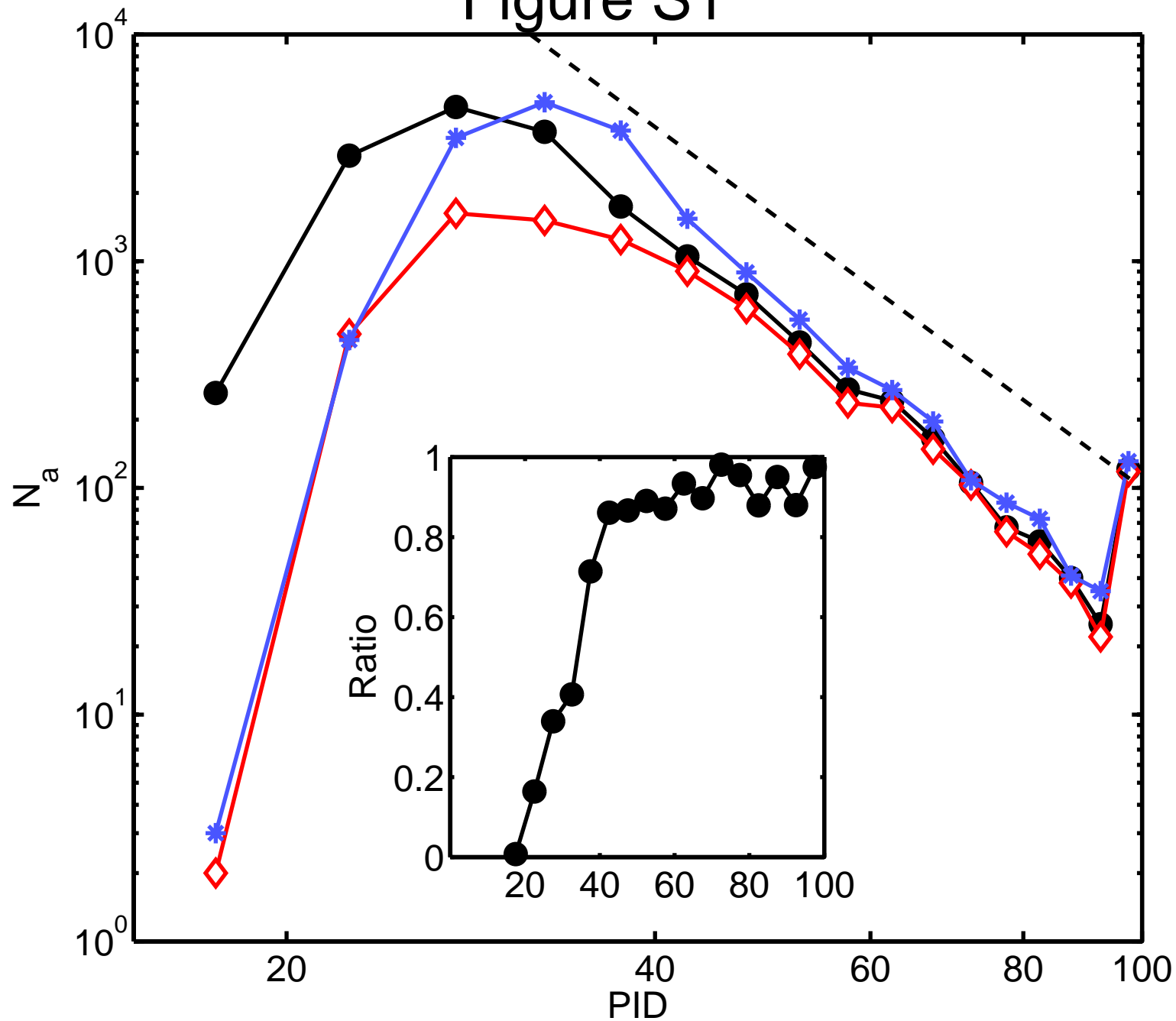

Supplement: Additional file 1 — The overall shape of the PID histogram is independent of the alignment algorithm and the E-value cutoff. The PID histogram Na(p) in the fly (D. melanogaster genomes when pairs of paralogous proteins were detected using the blastp algorithm [1] with E-value cutoff of 10-10 (filled circles) and 10-30 (open diamonds). The inset shows the ratio of these two histograms, which is very close to 1 for p > 40%. Thus the overall shape of Na(p) in most of the Region II (Fig. 1) is nearly + cutoff independent. The Na(p) also is insensitive to a particular algorithm used to align the pairs. Indeed, when paralogous pairs detected by the blastp with the E-value cutoff of 10-10 (filled circles) were realigned using the Smith-Waterman algorithm [28] the resulting distribution (blue stars) changed very little. [file 1745-6150-2-32-S1.pdf]

Figure S2

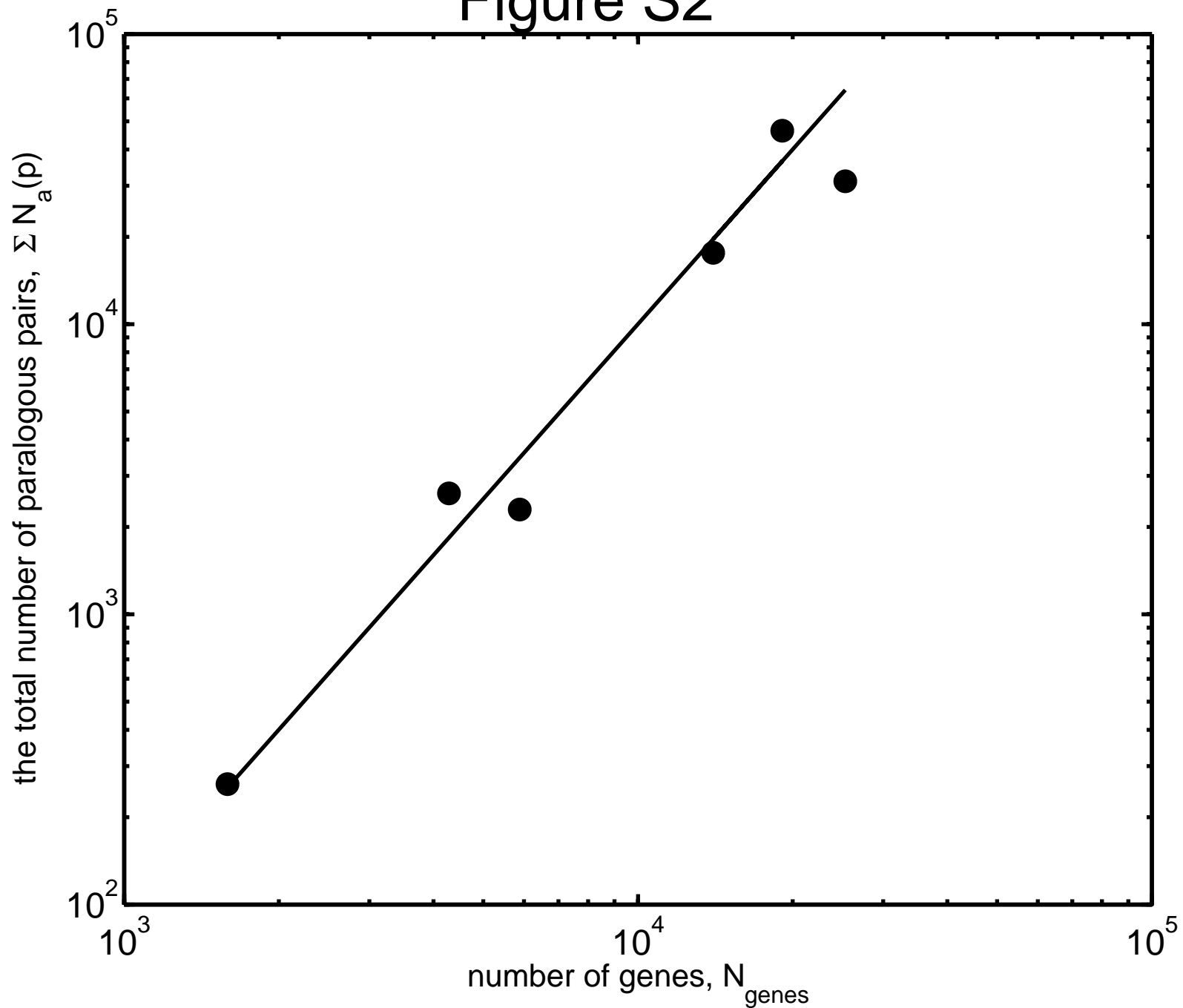

Supplement: Additional file 2 — The quadratic scaling of the total number of paralogous pairs with the number of genes in the genome. The total number of paralogous pairs ∑pNa(p) generated by the all-to-all alignment of all protein sequences encoded in the genome (the y-axis) scales as the square of the total number Ngenes of protein-coding genes in the genome. Solid symbols are six model organisms used in our study. The solid line has the slope 2 on this log-log plot. [file 1745-6150-2-32-S2.pdf]
